# The monkeypox virus: A public health challenge threatening Africa

**DOI:** 10.1002/puh2.33

**Published:** 2022-11-14

**Authors:** Emery Manirambona, Shuaibu Saidu Musa, Deborah Oluwaseun Shomuyiwa, Feruzi Abdu Salam, Okesanya Olalekan John, Daniel Garang Aluk Dinyo, Usman Abubakar Haruna, Alhaji Umar Sow, Don Eliseo Lucero‐Prisno, Kengo Nathan Ezie, Mohamed Samai, Lydia Aziato

**Affiliations:** ^1^ College of Medicine and Health Sciences University of Rwanda Kigali Rwanda; ^2^ Pan‐African Action for Health Equity Bujumbura Burundi; ^3^ Department of Nursing Science Ahmadu Bello University Zaria Nigeria; ^4^ Faculty of Pharmacy University of Lagos Lagos Nigeria; ^5^ Global Health Department Health Maintenance Organization in Africa Goma The Democratic Republic of Congo; ^6^ Department of Medical Laboratory Science Kwara State University Ilorin Nigeria; ^7^ Medical Department Médecins Sans Frontières‐France South Sudan Mission Juba South Sudan; ^8^ Department of Biomedical Science Nazarbayev University School of Medicine Nursultan Kazakhstan; ^9^ College of Medicine and Allied Health Sciences University of Sierra Leone Freetown Sierra Leone; ^10^ Department of Global Health and Development London School of Hygiene and Tropical Medicine London United Kingdom; ^11^ Faculty of Management and Development Studies University of the Philippines, Open University Los Baños Laguna Philippine; ^12^ Faculty of Public Health Mahidol University Bangkok Thailand; ^13^ Faculty of Medicine and Biomedical Sciences University of Garoua Garoua Cameroon; ^14^ University of Health and Allied Sciences Hohoe Ghana

**Keywords:** Africa, emerging virus, endemic, monkeypox, monkeypox diagnosis, monkeypox vaccine, public health challenge, zoonosis

## Abstract

On 23 July 2022, the World Health Organization reported monkeypox cases in over 75 countries and, as a result, declared the virus a Public Health Emergency of International Concern (PHEIC). Despite Africa being the centre of the zoonotic disease evolution, its healthcare systems have not provided optimum attention to the problem. The African healthcare system is under the threat of a repeat of the situation that transpired during the COVID‐19 pandemonium if stringent measures are not implemented immediately. Lessons learned from the COVID‐19 pandemic should inform emergency preparedness and response from African countries. Concerted and sustainable efforts can be achieved by reviewing and redesigning strategic preparedness plans for testing and vaccination as in previous pandemics. Africa needs to drive this response with collaboration and a united response, and with the use of strategic communication and a sense of urgency. Africa should prioritise access to available vaccines and invest in systems development for local vaccine production. In this article, we argue that monkeypox virus has serious public health implications in Africa and the region.

## INTRODUCTION

The Monkeypox virus (MPXV) is an enveloped double‐stranded DNA virus that belongs to the *Orthopoxvirus* genus of the *Poxviridae* family. This zoonotic viral infection has two distinct genetic clades: the central African (Congo Basin) clade and the West African clade [1]. The virus was first isolated in 1958 in a Danish laboratory from skin lesions of an imported macaque and was known to cause outbreaks in captive primates [2]. The first human case of MPXV was reported in 1970 in the Democratic Republic of Congo (DRC). Since then, numerous human cases of MPXV have been reported in the tropical rainforest areas of Western and other Central African countries. The most common mode of transmission was contact with wild animals such as rodents.

The MPXV outbreak is no longer limited to the endemic regions of Central and West Africa since 2003, when the United States of America reported its first human case. Between 1 January to 22 July 2022, there were 16,836 cumulative cases of Monkeypox reported in 75 countries worldwide [3]. This unusual quick spread of MPXV throughout all continents within such a short period has made it a global public health threat. Due to the rapid spread of the disease facilitated by international mobility and traffic, the World Health Organization (WHO) declared MPXV a Public Health Emergency of International Concern (PHEIC) based on International Health Regulations [4]. Unfortunately, there is a paucity of data to document the actual threat of the disease. This is surprising because the disease has been circulating on the continent for at least 50 years, thus reporting numerous cases and deaths. The lack of data may result from neglecting epidemics and lack of funds to conduct research to provide evidence on the nature of epidemics. Providing evidence remains crucial to implementing necessary strategies to stop the disease and improve public health preparedness [5]. As such, this paper aims to stimulate critical discussion and debate to fill some of the gaps in understanding the disease in Africa. The paper discusses MPXV as a public health concern in Africa and provides practical recommendations to policymakers.

### MPXV as a public health concern in Africa

MPXV has been an epidemic in some African countries for five decades. These countries include Benin, Cameroon and Liberia. The disease has also hit countries such as the DRC, the Central African Republic, and Nigeria. Other countries where it has been endemic are Gabon, Ivory Coast, South Sudan, and Sierra Leone. MPXV has also been endemic in Ghana, where it was only detected in animals.

According to the Africa Centres for Disease Control and Prevention (Africa CDC), recent MPXV outbreaks were reported in Cameroon, with one confirmed and 15 suspected cases in 2018. Other outbreaks were reported in Nigeria in 2017 ‐ the most populous country in Africa, and the Central African Republic in 2016. Interestingly, Nigeria reported over 200 confirmed and 500 suspected cases of MPXV in 2017. A 10% mortality rate was associated with the more virulent MPXV that infected many people in Central Africa [6]. Likewise, the DRC has been recording a high number of cases. In the past 10 years, thousands of suspected cases and hundreds of suspected fatalities have been related to MPXV in the DRC [6].

Although the current MPXV outbreak prominently spreads outside Africa, African countries are continuously under the threat of MPXV. Since January 2022, 1781 suspected and 250 confirmed cases and 75 deaths have been reported by Africa CDC as of 25 July 2022 [7]. This case fatality rate of 3.7% is alarming. It means that the disease has caused significant mortality. Considering the population at risk and the disease transmission, the overall disease burden and disability‐adjusted life year is likely to be significant in Africa.

The above numbers are significant to warrant immediate health system responses and action in all countries [7]. MPXV may have been infecting thousands and killing hundreds during COVID‐19 pandemic period because little attention was paid to it. However, measures such as travel restrictions, lockdowns and quarantine after international travel devised to curb the spread of COVID‐19 may have helped the spread of MPXV. Therefore, it is not surprising that MPXV has spread quickly worldwide since the lockdown measures were lifted in early 2022.

### Socio‐economic impact of monkeypox

The effects of outbreaks have devastating impacts on the vulnerable sections of the population [8]. They pose a significant risk to immunocompromised groups and families if immediate action is not taken. The healthcare system faces challenges related to diagnosis, treatment and disease prevention during the outbreak. The lives of health professionals are at significant risk especially when the health equipment supply chain is interrupted [9]. The ability of healthcare workers to provide care also decreases if they or their family members fall ill, as they would have to look after one another. The outbreak of MPXV could compound the burden on the health system that already faces multiple other issues.

Disease outbreaks of MPXV can lead to falling tax revenues and increased spending, leading to fiscal stress, particularly in lower‐middle‐income countries (LMICs) where fiscal constraints are high and tax systems need improvement. As such, an outbreak of MPXV could cause a short‐term fiscal and long‐term economic impact on nations. All efforts to contain the virus would cost an appreciable amount of resources, significantly impacting the national economy [9]. The population's productivity is drastically affected, thus impacting national economies and overall development. Labour shortages, transportation disruptions, job closures, and restricted trade and travel will hamper economic recovery from the effects of COVID‐19.

Outbreaks will have significant social and political impacts, such as population displacement, increased social tensions, discrimination, and unhealthy competition and protectionism among countries. It may cause severe demographic changes, moral shocks, and social and political unrest. Empirical evidence suggests that the MPXV outbreak may generate political tension and unrest, particularly among countries with weak socio‐policial and economic standing [9].

By taking the experiences from the COVID‐19 pandemic that has negatively impacted public finances, the monkeypox outbreak can cause similar effects if adequate and timely measures are not taken to prevent it. MPXV can cause the closing of economies and reduce lending opportunities among nations affected, which can depreciate the value of local currencies, making dollar‐denominated debt more challenging to repay [10]. Governments may also face budget deficits due to increased spending on social protection for unemployed and poor individuals and due to reduced tax revenues [11].

The MPXV outbreak is also likely to exacerbate the situation of gender inequality. Evidence has suggested the possible sexual transmission, mostly among men‐who‐have‐sex‐with‐men, based on the news reported in some countries. The need to understand the gender dimension of the virus transmission is imperative as it is known that women are far more affected than men during viral outbreaks and by their social and economic impact. They also bear the brunt of caregiving responsibilities if family members fall ill [12]. Similarly, the isolation of suspected and/or MPXV cases can lead to domestic violence with women as victim [13] resulting in job losses and economic instability [14].

### MPXV testing in Africa

Timely and accurate testing of MPXV can break the chains of transmission and curtail the outbreak. While the non‐endemic African countries recording cases, the region needs to boost MPXV surveillance by scaling up testing and revamping its testing capacity [15]. Establishing diagnostic capacity, as seen in the COVID‐19 response, is crucial to gaining knowledge for outbreak preparedness and response. With over 18,000 cases globally, Africa has just over 200 confirmed cases as of July 2022 [3]. Weak surveillance and lack of diagnostic capacity mean that cases are going undetected. The high number of suspected cases calls for the need to improve surveillance and diagnostic capacity [16].

The present testing process of the MPXV can be said to be complicated as lesions are required to be sampled for the testing. Test results can be obtained between 24 and 40 hours, depending on the sophistication of the testing laboratory. Samples in Africa are tested by sending first to a network of publicly‐funded Centres for Disease Control and Prevention Laboratory Network across countries. Rapid diagnosis is essential to optimise the testing process as it makes for impactful contact tracing for disease control. Updated machinery and equipment are required to reduce the processing time and optimise the diagnosis process. In a bid to improve the diagnostic process, the Africa CDC, in conjunction with the Nigeria Centre for Disease Control (NCDC), organised hands‐on training for 20 African Union member states on sample collection, isolation, interpretation and reporting of MPXV specimens using real‐time polymerase chain reaction (RT‐PCR) assay [17]. The training of health workforce on physical examination and history tracking and public engagement with widespread education on apparent signs and symptoms like high fever, skin eruptions and lymphadenopathy are important for identification of suspected cases.

With no vaccines in Africa and test kits in short supply, Africa needs to develop a strategy to prioritise mass testing in its response. While African countries have polymerase chain reaction machines available due to the COVID‐19‐driven, Africa needs new test kits, reagents and trainings to boost testing capacity. Testing can be rapidly scaled up with implementation as fully funded programs with optimal support from the international health community and development partners. While anticipated test volumes can be less than that during the COVID‐19 pandemic due to the lesser transmission rate, local disease surveillance networks deployed for COVID‐19 management can also be utilised for appropriate guidance and action. While the WHO undertakes the process of procuring thousands of monkeypox test kits for Africa [18], the WHO and the African countries must work to avoid a repeat of inequities in Africa's response to the outbreak.

### Monkeypox treatment and vaccination

Although no vaccine has been fully developed yet for MPXV, the smallpox vaccine has been seen as the optimally available option for containing the current outbreak. Despite the WHO declaration that places the MPXV outbreak on the highest alert level, the Africa CDC and the governments have not yet procured MPXV vaccine doses. This lack of vaccines in Africa provides the reality of limited resources for the region, serving the opportunities to call on action for those who can support vaccine programs in Africa. Africa CDC, stakeholders, the WHO, and others should find vaccine financing partners to ensure vaccine availability. To limit dependence on other countries for financing, Africa should find internal sources for its strategies by tapping internally sourced funds or using an Africa‐wide financing strategy. Donor aid should be tapped as a complement to locally sourced funds. This strategy would provide a good learning experience for the region as a form of preparation for future outbreak occurrences.

Countries such as Canada, the United Kingdom and the United States have begun implementing a ring vaccination strategy to curb the virus outbreak, which involves administering smallpox vaccines that are thought to be 85% effective against MPXV because the viruses are related [19]. In addition to the vaccine, an antiviral drug ‐ tecovirimat ‐ initially manufactured to treat smallpox, was licensed for use in monkeypox infection in 2022 [20]. The drug is also licensed to treat cowpox and infections caused by the *Orthopoxvirus* family. Interestingly, a prospective study conducted in the United Kingdom showed promising results from the use of tecovirimat [21] but it is nowhere to be seen in Africa.

MPXV is a public health challenge that led high‐resource countries to identify groups at high risk of transmission, thus prioritising them in vaccine and treatment. A global vaccine response completely eradicated the simimallpox virus that caused millions of deaths [22]. This approach would be crucial to stopping the MPXV transmission.

### Recommendations

Leveraging the lessons from the COVID‐19 response, Africa must rise strategically in resilience and collaboration to deal with the reemerging MPXV. African countries must reenact the best practices that prompted the response initiated against the COVID‐19 pandemic on the continent and rethink those actions which were not effective. Essential to the COVID‐19 response in Africa was the development of strategic preparedness and response plans considering the social, cultural, and political influences on the fragile African health systems. These plans should include providing technical recommendations and guidelines for the protection of the community and health workers, local and international engagement models, and the design and implementation of strategic efforts that support the sustainable management of the virus [23].

#### Information management

As the virus spreads to non‐endemic countries, focusing on strategic risk communication with accurate and adequate information to manage the spread is essential. Increased awareness improves public readiness and guides response and community actions such as minimization of physical contact and improved sanitation. Information on symptom identification and emergency and support services must be readily available. Africa must protect itself by the prevention and activation of public health measures. While these measures have gained appreciation with the COVID‐19 pandemic, open and constant information positioning is vital for continued participation and compliance. The continent must be vigilant against misinformation, fake news and infodemic, which all impacted the previous pandemic. With the virus affecting some unique populations in many countries, such as men‐having‐sex‐with‐men, packaging messages within Africa should be culturally sensitive, strategic and effective. Myths and other forms of wrong information should be properly addressed before vaccine hesitancy and complacency set in.

#### Preventive and public health measures

Coordinating global outbreak alerts and response networks include intensifying surveillance and public measures. Preventive and public health measures represent an important arm of infection containment. While quarantine and isolation are essential for disease management, present global guidelines do not recommend mobility restrictions for preventing disease transmission [24]. With changes in guidelines with increasing knowledge of the outbreak, standard risk reduction protocols will hold ground for safeguarding public health. Proactive environmental infection control should be integrated with the initiatives, including standard cleaning and infection procedures, especially in healthcare settings [24]. Source control will also include the prioritisation of transport and movement of patients as well as standard waste management practices. Elaborating and increasing the awareness and elaboration of physical distancing measures, personal protection and hygiene, as in handwashing and personal protective equipment [25] holds great value for public acceptance.

#### Case identification and management

Contact tracing and case identification should be followed by immediate actions such as appropriate laboratory investigation and clinical management. Increased investment in epidemiological investigations and research is essential in building knowledge on the virus and its evolution. While the continental focus has been on COVID‐19, the MPXV could prove just as deadly if neglected. The utilisation of data and innovative technologies is critical to Africa's response. Smart technologies and population data should be properly tapped to inform policy and practice immediately. Essential health services in African countries, especially the endemic regions, must be empowered for disease management and control. In addition to isolation as a management measure, provision of proper supportive care is critical. Optimal workforce capacity is crucial in the development of health system resilience.

#### Vaccination

Vaccination is an essential part of disease management. African countries should prioritise investment in vaccine manufacturing and acquisition [26]. Although COVID‐19 has brought to life the adaptability of the African continent to vaccine production and logistics, investment in the technology of people and systems to create an environment for vaccine production is essential. The evolution of the MPXV should attract investment in the research and development of safe and effective vaccines for the virus. The development of vaccination strategies is critical. While vaccine development can occur quickly, as seen with the COVID‐19 vaccine, Africa must be on the map for the available smallpox vaccines. This is not about requesting for help; it is about protecting the global community. This is attainable with collaborative efforts of the African countries to foster international engagement and push back the stigma directed toward the African public health system.

## CONCLUSION

The Monkeypox outbreak has turned into a significant global public health challenge as cases have been reported in many countries. This is particularly important in the African continent, especially in regions such as the DRC and other African countries where the virus has been confirmed to be endemic. The African healthcare system is again faces challenges as it is reminded of the impact of the COVID‐19 pandemic on the health and economy. Despite Africa being the epicentre of many zoonotic outbreaks, the healthcare systems are not given optimum attention until diseases, such as the MPXV, spill out of the continent and are detected in wealthier countries. Vaccines and medications usually developed to control and treat infections are mainly inaccessible to African countries. In light of these factors, the current MPXV outbreak will become a bigger threat to public health in Africa, further widening the health equity gap between African countries and the rich countries of the world. There is now an urgency to act upon this problem if the world has to prevent the further spread of the MPXV.

## AUTHOR CONTRIBUTIONS

Emery Manirambona, Shuaibu Saidu Musa, Deborah Oluwaseun Shomuyiwa: conceptualisation, project administration and design. Emery Manirambona, Shuaibu Saidu Musa, Deborah Oluwaseun Shomuyiwa,Feruzi Abdu Salam, Okesanya Olalekan John, Daniel Garang Aluk Dinyo, Usman Abubakar Haruna, Alhaji Umar Sow, and Kengo Nathan Ezie: data collection and literature review; preparation of the original draft and visualization. Emery Manirambona, Don Eliseo Lucero‐Prisno III, Mohamed Samai, Lydia Aziato: supervision, writing, reviewing, editing, and proofreading. All authors have read and confirmed that they meet ICMJE criteria for authorship.

## CONFLICT OF INTEREST

Emery Manirambona, Shuaibu Saidu Musa, Deborah Oluwaseun Shomuyiwa, and Don Eliseo Lucero‐Prisno III are Editorial Board members of Public Health Challenges and co‐authors of this article. They were excluded from editorial decision‐making related to the acceptance of this article for publication in the journal.

## FUNDING INFORMATION

No funding was received for the development of this paper.

## ETHICS APPROVAL

As this is a perspective article, no ethics approval was required.

## Data Availability

No database or primary data were used in preparing this manuscript.
